# A Cost-Effective Electric Vehicle Intelligent Charge Scheduling Method for Commercial Smart Parking Lots Using a Simplified Convex Relaxation Technique

**DOI:** 10.3390/s20174842

**Published:** 2020-08-27

**Authors:** Muhammad Jawad, Muhammad Bilal Qureshi, Sahibzada Muhammad Ali, Noman Shabbir, Muhammad Usman Shahid Khan, Afnan Aloraini, Raheel Nawaz

**Affiliations:** 1 Department of Electrical and Computer Engineering, CUI Lahore Campus, Lahore 54000, Pakistan; mjawad@cuilahore.edu.pk; 2 Department of Electrical and Computer Engineering, CUI Abbottabad Campus, Abbottabad 22060, Pakistan; hallianali@cuiatd.edu.pk; 3 Department of Electrical Power Engineering & Mechatronics, Tallinn University of Technology, 19086 Tallinn, Estonia; noshab@taltech.ee; 4 Department of Computer Science, CUI Abbottabad Campus, Abbottabad 22060, Pakistan; ushahid@cuiatd.edu.pk; 5 Department of Computer Science, Qassim University, Al Qassim 1162, Saudi Arabia; A.ALOURANI@qu.edu.sa; 6 Department of Operations Technology, Events and Technology Management, Manchester Metropolitan University, Manchester M15 6BH, UK; R.Nawaz@mmu.ac.uk

**Keywords:** intelligent charging, demand response, electric vehicle, linear programming, optimization, smart parking, smart grid

## Abstract

Deployment of efficient and cost-effective parking lots is a known bottleneck for the electric vehicles (EVs) sector. A comprehensive solution incorporating the requirements of all key stakeholders is required. Taking up the challenge, we propose a real-time EV smart parking lot model to attain the following objectives: (a) maximize the smart parking lot revenue by accommodating maximum number of EVs and (b) minimize the cost of power consumption by participating in a demand response (DR) program offered by the utility since it is a tool to answer and handle the electric power usage requirements for charging the EV in the smart parking lot. With a view to achieving these objectives, a linear programming-based binary/cyclic (0/1) optimization technique is developed for the EV charge scheduling process. It is difficult to solve the problems of binary optimization in real-time given that the complexity of the problem increases with the increase in number of EV. We deploy a simplified convex relaxation technique integrated with the linear programming solution to overcome this problem. The algorithm achieves: minimum power consumption cost of the EV smart parking lot; efficient utilization of available power; maximization of the number of the EV to be charged; and minimum impact on the EV battery lifecycle. DR participation provide benefits by offering time-based and incentive-based hourly intelligent charging schedules for the EV. A thorough comparison is drawn with existing variable charging rate-based techniques in order to demonstrate the comparative validity of our proposed technique. The simulation results show that even under no DR event, the proposed scheme results in 2.9% decrease in overall power consumption cost for a 500 EV scenario when compared to variable charging rate method. Moreover, in similar conditions, such as no DR event and for 500 EV arrived per day, there is a 2.8% increase in number of EV charged per day, 3.2% improvement in the average state-of-charge (SoC) of the EV, 12.47% reduction in the average time intervals required to achieve final SoC.

## 1. Introduction

The conventional transportation system is producing nearly 14% of total worldwide greenhouse discharges, which is estimated to increase further 50% by 2030 [1]. Air pollution alone is one of the major extraneous costs of transportation, especially as it directly influences the health of local inhabitants. The growing international desire for adopting environment-friendly technologies has resulted in the acceptance and usage of alternate fuel vehicles that can be hybrid and battery-operated electric vehicles (EVs). However, EVs have been unable to secure the confidence of customers and acquire a large market share yet. This is mainly due to their technical and infrastructure limitations, such as limited driving range and unavailability of charging facilities. Moreover, the resultant cost considerations also deter potential customers. In order to further the public acceptance of EVs, it is important to improve the EV infrastructure for all key stakeholders. 

A significant aspect of these infrastructure requirements is the abundant availability of charging points and charging stations. Charging places act as connection sources to the electrical grid for the EV, and the place of powering up the batteries for EV drivers. However, a major challenge for existing power systems will be to maintain demand response (DR) with the growing demand for electricity as a result of an increased number of EV parking lots. Currently, how to manage the adaptability of EV use and their charging without having a drastic impact on existing power grids is a contentious topic [2,3]. The recent advancements in control strategies and sensing visualize DR as an effective tool to help address the issue of demand-supply mismatch in electric power grids. The DR program provides customers with leverage to shift their electricity demand from peak hours to off-peak hours and as a consequence, derive benefits in terms of lower electricity prices and financial incentives. In Refs. [4,5,6], a number of DR programs were explored to lower the overall power usage of a household for load shifting and curtailing the appliances of residential households during peak hours. 

Modern utility grids need suitable candidates that can be involved in the DR programs for demand curtailment of the grid in peak hours. The large-scale EV parking lots are one of the most suitable solution due to following reasons: (a) by managing the accumulated electrical load of the parking lot can act as a large-scale demand curtailment player in the power market and (b) the electrical load flexibility of EV charging loads with adjustable and interruptible features can ensure that EV charge scheduling can coordinate with the grid’s DR programs to satisfy all charging demand of EVs, while fulfilling committed demand curbing for DR. In DR programs, the electricity tariffs are usually dynamic in nature and may include day-ahead market pricing [7,8], real-time pricing [9,10] or time-of-use structure of pricing [11]. Due to the adjustable features present in the EV charging load, it is possible for EV smart parking lots to explore possibilities to execute both incentive-based and price-based DR programs. 

Therefore, in this paper, a real-time charge management system (CMS) is developed to charge EVs parked in a smart parking lot by taking into consideration the advantages of DR programs. The EV charging mechanism is optimized using the linear programming (LP) and convex relaxation techniques. The objective of the CMS is to maximize the number of EVs that can be fully charged over a 24-h period and minimize the cost of energy consumption by participating in different DR programs. Moreover, the DR event will also curtail the total charging load of the smart parking lot. In the proposed CMS, the on-off (binary) charging strategy is employed to solve the EV charging problem while considering the state-of-health (SoH) of the EV battery and its C-rate. The C-rate is a measure of the rate at which a battery is charge/discharged relative to its maximum capacity. Under the binary charging technique, the EV attached to the charging pole will be charged at fixed maximum charging rate as per C-rate equals to 1 C. Thus, in addition to consuming less time, it will also have minimal impact on the battery health by fully utilizing the battery’s single charging cycle. However, the optimization of binary charging decision for every EV is a non-trivial task because the scheduling of EVs is formulated as a binary optimization task. The intelligent charging schedule can be determined using the generate and test method, also known as exhaustive search. However, it is not recommended for real-time implementation due to its computational expensive nature. Therefore, the problem is mitigated by developing a convex relaxation approach that is integrated with the LP, where near intelligent charging scheduling is computed. Finding the intelligent charging schedule for EV is a non-convex problem due to the high number of EV, and random arrival and departure times. Therefore, the binary constraint in the problem related to the on-off charging strategy is relaxed to solve the problem as a convex problem using LP. Thereafter, a modified mapping is used to convert the solution back to binary values. This whole process is named as the simplified convex relaxation approach integrated with the LP. More details about this process have been provided in Section 5. The main contributions of the paper are as follows:

Modelling of a realistic EV smart parking lot by considering: (a) regular and random arrival and departure times for EV; (b) different types of EV along with different battery capacities and charging rates; (c) level-2 battery charging standard; and (d) EV battery efficiency and life cycle.

- First, an on-off (0/1) charging technique is used for the EV, i.e., an EV selected for charging in a time interval will be charged at fixed maximum charging rate according to its level II charge rate. Charging an EV at constant power may extend the battery’s service time. Secondly, the communication overheads will be small as only a small subset of the EV will be required to contact and it would be more feasible to apply on/off charging scheme. However, the target is to charge maximum EV with the minimum charging price possible. Whereas, the scheduling of the EV for intelligent charging using the on-off/cyclic technique is a binary optimization issue that is computationally extensive to be resolved at run-time if the number of EV will increase. In order to address this issue, an EV charge scheduling technique named simplified convex relaxation approach integrated with the LP is utilized.
- The DR program can lower the cost of electricity for EV parking lots; therefore, DR events are introduced in the on-off charging technique. The on-off charging scheme not only respond to the variable electricity prices but also responds well for demand curtailment events from the grid.
- Second, a variable charging rate technique for the EV charging is tested while having fixed capacity limit of the EV charging station, i.e., all the charging poles in the EV parking lot are used to accommodate all arrived EVs. However, the drawbacks of variable charging rates are: (a) charging EVs at constant power could extend the service time of the battery, which is a disadvantage in variable charging rate [12] (b) variable charge rate will extend the charging time of the battery.
- A thorough comparison of both binary charging scheme and variable charging scheme is conducted based on maximum revenue generation, energy consumption cost, number of EVs charging in a daytime, and the impact on the battery life of the EVs.

The paper is organized as follows: A detailed literature review is presented in Section 2, the system model and optimization technique are discussed in Section 3, Section 4 comprises of results, discussions, and comparative analysis. Finally, the paper is concluded in Section 5 along with some proposals for future research directions.

## 2. Literature Review

In the past, researchers have developed EV smart parking lot models and applied numerous optimization techniques in order to schedule EV charging. The objectives of all such EV scheduling techniques in EV smart parking lots are at least one of the following: (a) maximize the number of EVs charged in an allocated time [13]; (b) maximize the smart parking lot profit [14]; (c) minimize the EV owner’s charging cost [15]; and (d) minimize the peak demand by participating in DR [16]. 

In Refs. [13], the authors address the problem of EV charging schedules from the perspective of smart parking lot operator and EV owners. Through the utilization of quadratic problems, the optimization objectives were to maximize the number of EVs charged and the overall revenue of the smart parking lot while minimizing the EV charging costs for the EV owners. Similarly, in Ref. [17], game theory is used to schedule the EV charging to maximize the utilization of the smart parking lot by increasing the number of EVs to be charged. Accordingly, a greater number of EV owners can be accommodated. However, the authors have not considered the stochastic features of electricity price variations and the driving patterns of the EV. In Refs. [18,19], MILP and fuzzy linear programming (FLM) are used, respectively, to maximize the smart parking lot profit by effectively optimizing the charging schedule of the EVs. Similarly, in Ref. [20], a linear programming (LP) technique and dynamic programming (DP) model are used, respectively, to maximize the smart parking lot profit and to minimize the charging costs of EV owners. The problem with LP is how to simultaneously handle both real number and integers; therefore, MILP is more suitable then LP. The DP lacks a general formulation and every problem need to be addressed in its own way. Moreover, the DP consumes more memory while storing the results of intermediate steps, which is not the case in MILP. 

The DR program is an effective tool to minimize the cost of energy consumption while participating at a different level of programs. Therefore, in Ref. [14], the authors introduced the participation of EV smart parking lots in incentive-based and price-based DR programs along with the stochastic programming optimization technique, with a view to maximizing the EV smart parking lot profit. A similar technique with same the objective is used in Ref. [21] wherein the authors introduce smart parking lot as an aggregator agent in the real-time DR market. The energy source is parked EVs in the smart parking lot. Therefore, in Ref. [22], the EV smart parking lot is introduced as a multi-energy system (MES) to enhance the profit of the smart parking lot and to improve the operational capability of the MES. 

Meanwhile, a graded control algorithm for EV charging across several aggregators is proposed in [11] to minimize the peak load of the smart parking lot and electricity cost. The heuristics are developed in such a manner that at first, the distribution system operator (DSO) will solve the charging curve by participating in the DR program, after which the charging power will be allocated to each EV. An algorithm based on quadratic programming (QP) is proposed in Ref. [15] to optimize the charging schedule of EVs in order to minimize the charging cost of EV owners. Similarly, the DP is used in Ref. [23] for EV charging schedules with an aim to maximize the smart parking lot profit and minimize the EV owner’s charging cost. In Ref. [23], the DP is solved by assuming the parked EVs in the smart parking lot as an aggregated battery bank. A simulation platform named Okeanos is proposed in Ref. [24] based on a multi-agent DR program with an aim to get benefits from the optimal EV charging. According to the authors, increasing the number of charged EVs and minimizing the electricity tariff through the DR program can help optimize the EV charging schedule [24]. In Ref. [25] the authors developed an algorithm to minimize the EV owner’s charging cost by combining a distributed DR method and parked EVs as a storage capability. Similarly, using the LP algorithm, a distributed DR method using the random usage pattern of EVs is proposed in Ref. [26]. The objective of the model is to minimize the peak demand of the utility grid to minimize electricity cost. In Ref. [27], a real-time EV charging scheme for EV smart parking lot is proposed using MILP that coordinates and priorities requirements of EV charging and discharging powers with the power generation of the utility grid, renewable energy sources (RES), energy storage system (ESS), and electricity price preferences. 

In Ref. [28], a distributed EV charge management scheme is proposed from the perspective of EV owners to minimize the wait time of EV charging on parking lots. The authors proposed a P/S communication framework to utilize charging reservation effectively. In Ref. [29], another similar work, the authors proposed a preempted charging recommendation system for the income EV using V2V based reservation system with an aim to minimize on-the-move charge time and travelling time. In our work, we incorporate the arrival and departure time of the EV to ensure no delay in service for EV charging, while the advance reservation system for EVs does not lie in the scope of our work. 

To the best of our knowledge and analyzing the previous studies described in this Section, the proposed work is comprehensive in that the proposed scheme aims to use EV smart parking lots as a service provider and a decision-maker in DR program to optimize the intelligent charging schedule of parked EV. We developed an objective function that maximizes the number of EVs charged at a given time. However, the selection process of EV charging involves EV charging priority, state-of-charge (SoC), and electricity pricing preference; therefore, the advantages of the objective function are manifold, such as maximization of the smart parking lot profit, minimization of the EV charging cost for the EV owners, minimization of the peak demand by participating in different DR programs, and minimized impact on the state-of-health (SOH) of the EVs’ battery by charging the battery at the maximum and fixed maximum charge rate as per level-II charging of EV at C-rate equals 1 C.

## 3. System Model

**Figure 1:**
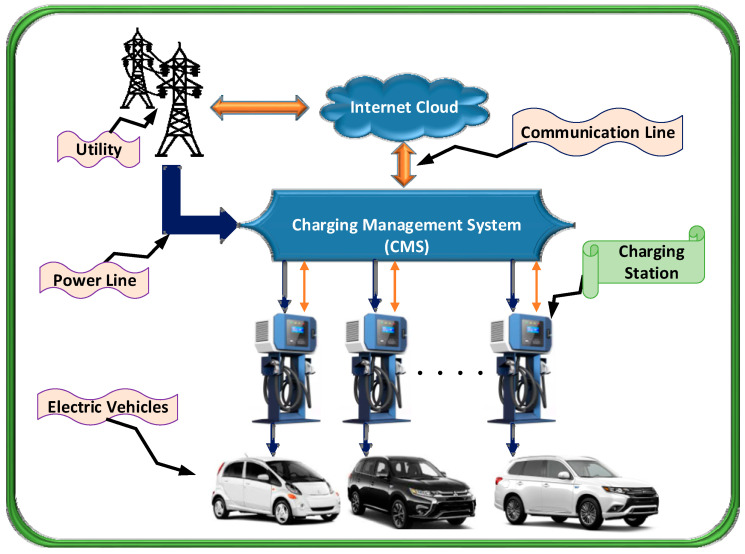
Charging Management System (CMS).

The system model for charging EVs in the EV smart parking lot is depicted in Figure 1. The model includes the following: (a) main grid (utility grid); (b) the aggregated electric load of parked EV; (c) charge management model (CMS); and (d) real-time DR power market. The primary source of energy for the EV smart parking lot is the main grid. The LP and simplified convex relaxation techniques are used to optimize the EV’ charge scheduling.

### 3.1. Preliminary Discussion

In the future, as the demand for EV charging increases, smart parking lot operators will be encouraged to install more charging points to incorporate more EVs. However, charging power capacity is a hard constraint that will limit the number of EVs to be charged at a given point in time. Moreover, variables, such as arrival time, departure time, EV battery capacity, driving cycle, and maximum charge rate of any EV depend on the vehicle type and manufacturer. Therefore, an optimized EV charging strategy is needed to control and manage the charging capacity of the smart parking lot in order to provide charging services to a large number of customers by taking into consideration the departure time of the EVs. Moreover, for the purpose of avoiding maximum capacity overload, a charging priority needs to be determined for the parked EVs to ensure fair charging preferences and to provide enough charging for an EV before departure time. Charging all the parked EVs simultaneously can overburden the utility grid due to the maximum power consumption capacity cap imposed on the smart parking lot. However, by using an intelligent EV charge scheduling algorithm, the peak load demand can be managed, delayed, or optimized by taking into consideration the departure time of the EV. This technique can make EV smart parking lots good candidates as aggregated agents in the DR program. Therefore, the EV smart parking lot will be suitable for the day-ahead electricity pricing tariff by managing their electric load throughout 24-h. Considering all the aforementioned attributes, the proposed CMS is designed in a manner that can optimize the EV charging schedule by managing the request of the DR program using a day-ahead electricity pricing tariff.

As illustrated in Figure 1, the CMS establishes two-way uninterruptable communication between the utility grid and the smart parking lot by sharing the electricity load curve with the grid and optimizing the scheduling of the parked EVs in accordance with the DR requirement of the grid. Moreover, the CMS computes the total electricity consumption cost. Therefore, the objective of the CMS is to manage the DR curve for the smart parking lot. The CMS stores the arrival time of each EV parked at the charging station to optimize the charging schedule of each parked EV. Moreover, the driver needs to enter the departure time and charge ranking on the smart parking station pole. Furthermore, the CMS computes the charge rate for each parked EV as the maximum charging rate varies for different make and model of the EV. Therefore, the idea behind the optimized CMS is to charge the parked EVs at fixed charge rates or halt the charging considering the departure time of the EVs. In this process, there must be an increase in the count of the number of fully charged EV during the whole day.

The motivation to participate in the DR program initiated by the utility grid is the demand flexibility of the charging loads (EVs) in the smart parking lot. The DR programs can be either fixed or time varying. The demand reduction curve programmed by the smart parking lot relies on the DR proceedings that include the fruitful bids processed in the day-ahead environment for the bidding of the required demand profile.

### 3.2. EV Charge Scheduling Technique

Let P denote the number of charging poles to offer charging facility for the EVs that have arrived in the EV smart parking lot. The CMS will develop a real-time (0/1) cyclic optimized charging schedule technique for the EVs attached to the P charging poles. The arrival and departure of the EVs in the smart parking lot is a continuous process; however, a sampling interval $S_{t}$ is taken for the decision-related propose of the real-time algorithm. The CMS will optimize the EV charging schedule after every $S_{t}$ time interval. Therefore, the 24 h in a day are divided into X time intervals such that $X=T/S_{t}$. If an EV is attached to the pth charging pole at a real-valued time interval $t_{p}^{arr}$, an enrolling status will appear, and the charging pole is considered as being triggered. A binary variable $\delta_{p}^{x}$ will determine the connection state for each pth charging pole at the xth time interval, where p represents the number of charging pole p=1,…,P and x is the number of time interval x=1,…,X. The variable $\delta_{p}^{x}$ = 1 if the pth charging pole is connected to an EV; otherwise $\delta_{p}^{x}=0$. Given that the daytime is divided into X time intervals, the arrival time interval of the EV attached to the pth pole is computed as: $t_{p}^{arr}$/$S_{t}$. A rounding with ceiling operator ⎡.⎤ is applied to select the lowest integer value as $n_{p}^{arr}=⎡t_{p}^{arr}/S_{t}⎤$. The driver is bound to insert the departure real-valued time. Similarly, let the departure time provided by the driver at the pth charging pole be $t_{p}^{dep}$. The departure time interval of the EV attached to the pth pole is computed as: $n_{p}^{dep}=⎣t_{n}^{dep}/S_{t}⎦$. The floor rounding operator ⎣.⎦ is used to select the highest previous integer value. Meanwhile, a binary variable $k_{p}^{x}$ is used to record the charging status of the attached EV. If an EV attached to the pth pole in time interval x is charging, the variable $k_{p}^{x}$ = 1; otherwise $k_{p}^{x}$ = 0. However, if $\delta_{p}^{x}$ = 1, then the $k_{p}^{x}$ can have values 0 or 1 depending on whether or not EV is charging, but if $\delta_{p}^{x}$ = 0, then definitely the $k_{p}^{x}$ = 0. All the notations used in the system model are defined in Table 1. 

As stated earlier, the purpose of the EV charging scheme is to maximize the number of EVs that have been charged during the course of the entire day and to minimize the electricity cost paid to the utility grid by participating in the DR program. Moreover, a valid charging priority scheme is integrated with the optimization problem along with an electricity preference price. 

### 3.3. Electric Vehicle Charging Priority and Preference

A weighting parameter is formulated in order to compute the charging priority of an EV attached to the p-th charging pole. The parameter contains the capacity of the p-th pole to refill the EV battery and the remaining time to charge the battery. The EV battery’s state-of-charge (SoC) attached to the p-th pole in time interval x is denoted as $\gamma_{p}^{x}$ and $C_{p}^{cap}$ represents its battery capacity. Therefore, the charging time intervals required to charge the EV is computed as follows at the x-th time interval:

(1) $$U_{p}^{x}=n_{p}^{dep}-x.$$

We define a variable rank function $\omega_{p}\in \left[0,1\right]$ for every EV that comes for charging at the smart parking lot. Although a higher rank will be given to the executive customers, they will be paying higher membership fees. Therefore, the weighted charging priority of every EV attached to the pth charging pole during the xth time interval is computed as follows, irrespective of its been charged or not:

(2) $$r_{p}^{x}=\{\begin{array}{c} \frac{\omega_{p}C_{p}^{cap}\left(\gamma^{max}-\gamma_{p}^{x}\right)}{Z_{p}^{max}U_{p}^{x}},\;if\;\delta_{p}^{x}=1;\\ 0,\;if\;\delta_{p}^{x}=0. \end{array}$$

In Equation (2), the term $\omega_{p}C_{p}^{cap}\left(\gamma^{max}-\gamma_{p}^{x}\right)$ represents the battery capacity that needs to be filled during the stay of the parked EV up to a maximum limit of the SoC $\gamma^{max}$. This term also implies that an EV with a lower SoC will have greater needs for charging. In the proposed work, if an EV attached to the p-th charging pole is selected to be charged, then it will be charged at the maximum charging rate $Z_{p}^{max}$ of that EV. Therefore, the denominator term of the Equation (2) denotes the maximum charging energy that is provided to the EV. If the value of $U_{p}^{x}$ is low, the EV must be charged on priority before the possible departure time. The charging priority in Equation (2) is a normalized factor ($r_{p}^{x}\in \left[0,1\right]$) because the nominator is divided by a maximum value. If an EV departs from the smart parking lot after the charging process, then the p-th charging pole is set to be free by setting $\delta_{p}^{x}=0$ along with weighted charging priority factor $r_{p}^{x}=0$. The charging pole will be re-activated again by setting $\delta_{p}^{x}=1$, if another EV arrives and re-attaches to the p-th charging pole and its corresponding weighted charging priority factor $r_{p}^{x}$ will be computed again using Equation (2). 

It is necessary to manage the charging demand of the parked EVs within the desired time period; however, maximizing the smart parking lot profit is another important factor that must not be disregarded. Therefore, maximizing the electricity bill by utilizing dynamic electricity pricing is another important factor. Moreover, the maximum number of EVs should be charged at the time of low electricity pricing to minimize the power consumption cost. In order to model these factors, we assume that $\beta_{max}$ and $\beta_{min}$ are the highest and lowest rates of electricity, respectively quoted by the utility grid for the EV smart parking lot. Therefore, the $\alpha_{p}^{x}$ is defined as an additional parameter to represent the preference level of the pth charging pole to charge the associated EV for the electricity rate $\beta^{x}$ at the xth time interval. The parameter is defined as:

(3) $$\alpha_{p}^{x}=\frac{\left(\beta_{max}-\beta^{x}\right)}{\left(\beta_{max}-\beta_{min}\right)},\;\forall \;p=1\ldots P,\;x=1\ldots X.$$

In Equation (3), the value of the parameter $\alpha_{p}^{x}$ will be high when the electricity price is low and vice versa. The denominator term is used to normalize the preference level as $\alpha_{p}^{x}\in \left[0,1\right]$.

## 4. Optimization Technique for Cyclic (On-Off) Scheduling of Electric Vehicles

The purpose of the EV’ charge scheduling technique is to manage the charging of all attached EVs while keeping the maximum power consumption of the smart parking lot under maximum permissible demand limit. The EVs selected for charging at any time interval x is based on the weighted charging priority parameter $r_{p}^{x}$ and the preferred electricity rate parameter $\alpha_{p}^{x}$. We defined a set I={1,…,P} to record the indices number of all the charging poles connected to an EV at the time interval x while compiling a set $\emptyset^{x}$ = $\left\{r_{1}^{x},r_{2}^{x},\ldots ,r_{P}^{x}\right\}$ that contains the record of the weighted charging priority parameters of every EV attached to the charging poles at the x-th time interval. The purpose of this record is to evaluate each charging pole one by one to observe the preference level of each attached EV and prioritizing the EV with a high weighted charging priority number, while keeping the total charging demand below the maximum available capacity limit, including the curtailment of DR demand. 

The purpose of the proposed optimization technique is to find the optimized set of EV to be charged at xth time interval. Therefore, a descending order operator (Sort(.)) is applied to the set $\emptyset^{x}$ along with its indices set I. The sorted charging pole indices with regard to the descending weighted charging priority parameters are stored in a new set $\Pi^{x}$. Therefore, we assign the highest priority to the charging pole having the highest $r_{p}^{x}$ value because the charging poles indices set $\Pi^{x}$ are rearranged in the descending order and the poles will be selected for charging until the limit of power capacity is reached. The set $\Pi^{x}$ is defined as:

(4) $$\Pi^{x}=Sort\left(I\right){|}_{\emptyset^{x}}$$

The optimization of the EV charge scheduling is to enhance the number of EVs charged in a given time interval. At the present q-th time interval, an objective function needs to be maximized that is described as the product of weighted charging priority parameters $r_{p}^{y}$ and electricity price preference level $\alpha_{p}^{y}$. The expression for the objective function is formulated as:

(5) $$\underset{k_{p}^{y},x=q,..,X}{\max}\sum_{y=x}^{X}\sum_{p\in \Pi^{x}}k_{p}^{y}r_{p}^{y}\alpha_{p}^{y}$$

In Equation (5), the binary variable $k_{p}^{x}$ represent the binary parameters to be optimized. The ideal EV charge scheduling is intended to increase the count of the EVs selected for charging at the given time interval as well as to reduce the electricity cost depending on the variable electricity rates governed by the utility. While the EV attached to the p-th charging pole of the smart parking lot will occupy the charging pole as per time intervals $x\;\in \;\left[n_{p}^{arr},n_{p}^{dep}\right]$, the optimization of the EV charging arrangement can be performed from the present q-th time interval till the day ends, such that x ∈ [q,X] to ease the calculation. This is because it was evident that $r_{p}^{x}=0,\;\forall \;x\;\in \;\left[n_{p}^{dep},X\right],$ as mentioned in Equation (2)

Suppose $Z_{total}$ is the total power capacity bound offered to the smart parking lot for the EV charging process, excluding the DR program, and let $Z_{DR}^{x}$ be the demand curbing for the DR event at the given x-th time interval. The overall charging requirement is controlled by the demand boundary limit $\left(\;Z_{total}-Z_{DR}^{x}\right)$ at the given x-th time interval, such as:

(6) $$\underset{p\in \Pi^{x}}{\sum }k_{p}^{x}Z_{p}^{max}\leq Z_{total}-Z_{DR}^{x},\;x=q,\ldots ,X.$$

It is ensured that the minimum energy requirement of an EV for the next travelling is fulfilled. Therefore, the charging state for the EV being charged by the p-th charging section at the given x-th time interval is controlled by the lower boundary $\gamma^{min}$. Moreover, the SoC of the EV is constrained by the upper boundary $\gamma^{max}$ to control overcharging. Assume η denotes the EV charging efficiency; therefore, the constraints applied on the charging is written as follows:

(7) $$\gamma_{p}^{x}C_{p}^{cap}+\eta Z_{p}^{max}k_{p}^{x}S_{t}\geq \gamma^{min}C_{p}^{cap},\;x=q\ldots X;$$

(8) $$\gamma_{p}^{x}C_{p}^{cap}+\eta Z_{p}^{max}k_{p}^{x}S_{t}\leq \gamma^{max}C_{p}^{cap},\;x=q\ldots X;$$

Finally, the SoC of each EV’s battery for the next time interval is calculated as:

(9) $$\gamma_{p}^{x+1}=\gamma_{p}^{x}+\frac{\eta Z_{p}^{max}k_{p}^{x}S_{t}}{C_{p}^{cap}}\;$$

## 5. Simplified Convex Relaxation Methodology

The binary optimization function is derived in Equation (5) under the inequality constraints Equations (6)–(8) and SoC update rule defined in Equation (9). In Equation (5), the binary variables include $k_{p}^{x}\;\in \;\left\{0,1\right\},\;p=1\ldots P,\;x=1\ldots X$; therefore, solving Equation (5) is a binary optimization problem. However, the nature of binary search optimization is extensive and influenced by the imprecation of dimensionality. Moreover, binary search is more complicated than linear search as it overkills for a very small number of variables/elements or provides an infeasible solution for an oversized set of variables, such as if the number of time slots X or the number of charging pole P. Furthermore, the list of the variables needs to be sorted to use the binary search algorithm, which is often unfeasible, specifically for the case when the number of variables is constantly increasing. In addition, the binary search algorithm only works for less-than inequality constraints. Therefore, based on multiple testing, we concluded that the binary optimization problem defined in Equations (5)–(8) is not solvable in real-time for the EV charging schedules. To address the aforesaid issue a simplified convex relaxation technique is applied in this paper. The simplified convex relaxation technique is a two-step process. First, the decision variable $k_{p}^{x}\;\in \;\left\{0,1\right\}$ of Equation (5), which is binary in nature, is now relaxed ${\hat{k}}_{p}^{x}\;\in \;\left[0,1\right]$ to allow the decision for the charging is real-valued. Therefore, the LP is used to optimize the approximated newly defined optimization problem as follows:

(10) $$\underset{{\hat{k}}_{p}^{x},x=q,..,X}{\max}\sum_{y=x}^{X}\sum_{p\in \Pi^{x}}{\hat{k}}_{p}^{y}r_{p}^{y}\alpha_{p}^{y}$$

The constraints for the objective function are defined as follows:

(11) $$\sum_{p\in \Pi^{x}}{\hat{k}}_{p}^{x}Z_{p}^{max}\leq Z_{total}-Z_{DR}^{x},\;x=q\ldots X.$$

(12) $$\gamma_{p}^{x}C_{p}^{cap}+\eta Z_{p}^{max}{\hat{k}}_{p}^{x}S_{t}\geq \gamma^{min}C_{p}^{cap},\;x=q\ldots X;$$

(13) $$\gamma_{p}^{x}C_{p}^{cap}+\eta Z_{p}^{max}{\hat{k}}_{p}^{x}S_{t}\;\leq \;\gamma^{max}C_{p}^{cap},\;x=q\ldots X;$$

Computationally, LP is an efficient technique and does not diverge with the increase in system dimensionality and size. Therefore, we found LP more suitable for the scheduling of EV charging in real-time in our application as all variables are linear [30]. However, a problem arises as to the relaxed variable ${\hat{k}}_{p}^{x}$ have fractional value output after LP optimization. With regard to the proposed on-off charging strategy, for EV charging needs to convert back the fractional values of the ${\hat{k}}_{p}^{x}$ to binary values 0 and 1. Therefore, in second step. A precise mapping is used to convert ${\hat{k}}_{p}^{x}$ ∈ [0,1] to $k_{p}^{x}\;\in \;\left\{0,1\right\}$. Moreover, the constraints defined in Equations (6)–(8) also need to be ensured by the mappings. In order to map fractional values to binary values, the charging poles indices set $\Omega =\left\{1,\ldots ,P_{y}\right\}$ is computed such that all the indices of pole having associated ${\hat{k}}_{p}^{x}$ values between 0 and 1 are included in set Ω, where $P_{y}\leq P$. Moreover, a new set is defined $\Gamma^{x}=\left\{{\hat{k}}_{1}^{x},{\hat{k}}_{2}^{x},\;\ldots ,\;{\hat{k}}_{P}^{x}\right\}$ to store the values of the variable $k_{p}^{x}$ for all the charging poles at the x-th time interval. The indices set Ω and variable set $\Gamma^{x}$ are used to apply descending order operation on the set $\Gamma^{x}$, such as:

(14) $$\Lambda^{x}=Sort\left(I\right){|}_{\Gamma^{x}}$$

In order to generate $k_{p}^{x}$ all such poles having ${\hat{k}}_{p}^{x}=1$ are automatically selected in the matrix $k_{p}^{x}$. The remaining assignment of binary values in the matrix $k_{p}^{x}$ involves the satisfaction of Equation (15).

(15) $$\sum_{p\in \Lambda^{x}}{\hat{k}}_{p}^{x}Z_{p}^{max}\leq \;\;Z_{total}-Z_{DR}^{x},\;x=q,\ldots ,X.$$

At each time interval x, the SoC of each EV is calculated using Equation (9) once the $k_{p}^{x}$ is computed. 

## 6. Results and Discussions

### 6.1. Simulation Settings

In our simulations, we used P=200 charging poles in the EV smart parking lot in order to estimate the effectiveness of the proposed work. The overall time period T is said to be 24 h for the EV charging and sampling time to measure the best and ideal EV charging scheduling, wherein $S_{t}$ is fixed to 10 min. Therefore, the overall time intervals are given by X=TSt=144 min. The SoC for every EV must have a defined higher ($\gamma^{max}$) and lower ($\gamma^{min}$) limits, selected as 0.99 and 0.66, respectively [31]. Moreover, the day-ahead electricity pricing tariffs of Houston (TX, USA) for the year 2019 are used in the simulations [30].

EV arrival at the smart parking lot can be regular or random in nature; therefore, both types of EVs are taken into consideration in the simulations to present more accurate designing of the EV smart parking lot, where the ratio for former to later is 6:4. The EVs having a regular presence in the smart parking lot can be easily predicted owing to their comparatively fixed entering and leaving times. A typical Gaussian model layout is used to generate synthetic data for both the entry and exit time of the regular EV. The arrival time of the regular EV is generated using normal distribution having mean ($\mu_{arv\;}$) and standard deviation ($\sigma_{arv\;}$) as 6:00 am and 60 min, respectively, and is given by:

(16) $$f\left(x\right)=\frac{1}{\sigma \sqrt{2\tau }}e^{-\frac{1}{2}{\left(\frac{x-\mu }{\sigma }\right)}^{2}}$$

The leaving times of the EV are also generated using the normal Gaussian distribution, where $\mu_{dep},$ and $\sigma_{dep}$ are 5:00 pm and 120 min, accordingly. In the wake of unforeseeable visits, the random EV have greater uncertain habits of utilizing the EV charging station. In order to simulate the irregular consumption habits, the arrival and departure time of random EV are equally distributed across the scheduling interval x ∈ [1,X] and the probability density function for gaussian uniform distribution is given by:

(17) $$f\left(x\right)=\{\begin{array}{c} \frac{1}{2\sigma \sqrt{3}}\;for-\sigma \sqrt{3}\leq x-\mu \leq \sigma \sqrt{3}\\ 0\;otherwise \end{array}$$

**Figure 2:**
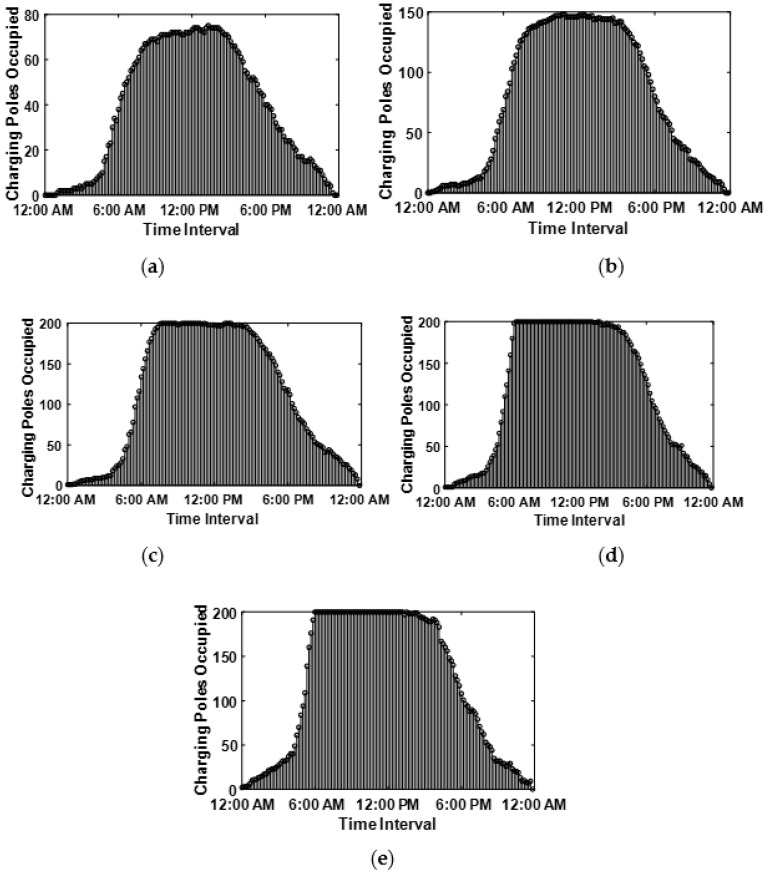
Number of Charging Pole Captured when (**a**) E=100, (**b**) E=200, (**c**) E=300, (**d**) E=400, and (**e**) E=500.

Suppose E is the number of EVs arriving at the smart parking lot for charging during the whole day which is, for the intervals x=1,…,X. It is possible that any charging pole can be utilized more than once to charge EVs throughout the day. Therefore, the charging pole can be reused instantly after the admitted car finishes charging and departs from the smart parking station. The simulation is conducted to model and observes different frequencies of EV arrival and for this purpose we used the following settings for the number of EVs entering the smart parking lot during the whole day E=[100–500] with a step of 100. However, the smart parking lot has limited charging poles that are selected as P=200; for this reason, the variable count of EV are simulated for the time interval x=1,…,X. The probability density function of a different number of EV (E) over the whole day is plotted in Figure 2, which illustrates the occupied charging poles in each time interval. Notably, as soon as the E increases to 300 and above, the charging station seems full for many time intervals given that all 200 charging poles are occupied.

The electrical characteristics of recently manufactured EV and their usage in percentage in the simulations are given in Table 2 [32]. Moreover, the level 2 battery charging mechanism is adopted in the simulations [33]. The initial SoC of an arrived EV is considered as being equally distributed in the interval [0.1–0.4], whereas charging efficiency η of each EV is set to be 0.9 [34]. The amount of EV with membership levels of low, average, and high is set to be 20%, 50% and 30% of total numbers of EV (E), respectively. The overall capacity threshold for the entire smart parking lot is set as $Z_{total}=500$ kW. All simulations of our proposed model are carried out on a server SYS-7047GR-TRF system using MATLAB optimization toolbox.

### 6.2. Simulation Results of Proposed On-Off Charging Scheme

**Figure 3:**
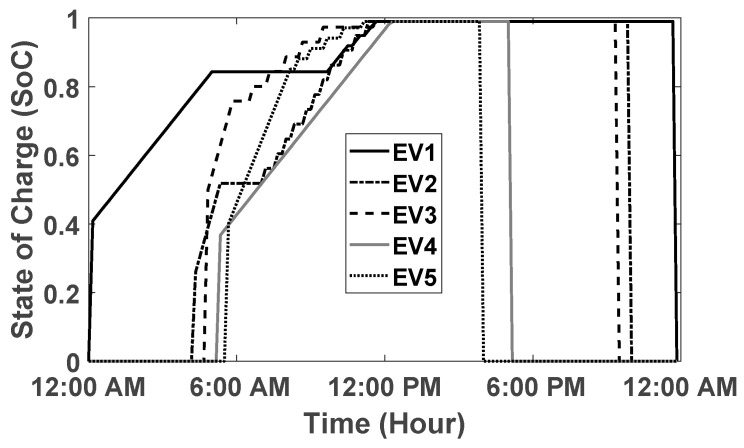
State of Charge of Randomly Selected EV.

**Figure 4:**
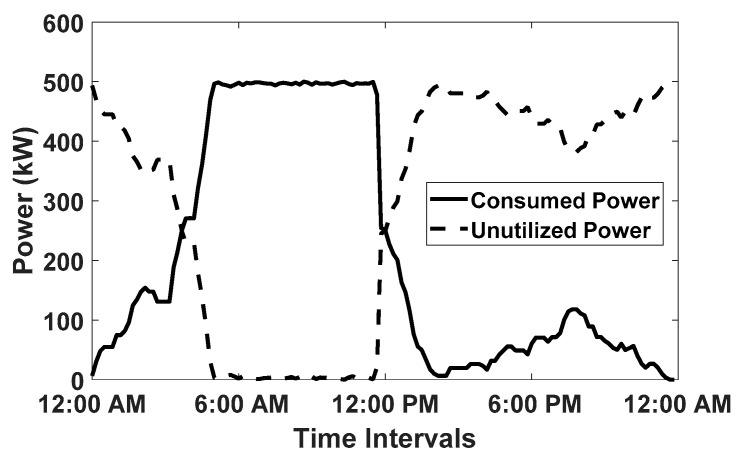
Consumed and Unutilized Power in 24 h, when E=500.

**Figure 5:**
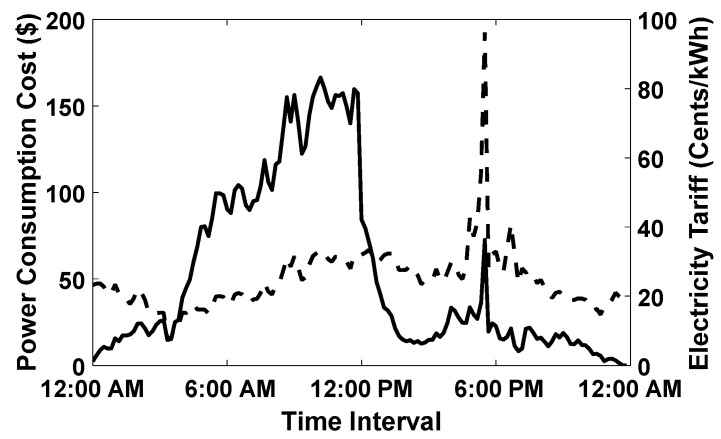
Power Consumption Cost and Electricity Tariff in 24 h, when E=500.

The goal of the proposed algorithm is to fully charge any EV that arrives at the smart parking lot. Since the SoC of each EV is observed before departure; therefore, the SoC of randomly selected five EVs is plotted in Figure 3. In Figure 3, it can be observed that the EVs leaving the smart parking lot are fully charged (SoC=1). In addition, it is evident that the SoC of the EVs is gradually increasing with charging in each time interval x. Similarly, In Figure 4, the consumed and the remaining power of the grid is plotted for the scenario when E=500. The Figure 4 provides evidence that during the working hours, the overall capacity threshold for the entire smart parking lot reaches its limit and many newly arrived EV must wait in a queue before getting an opportunity to be charged. Moreover, the available power is underutilized for many time intervals. Furthermore, the Figure 5 shows that the overall charging cost of the entire smart parking lot is computed separately in each time interval using the number of EV that have been charged in that slot multiplied with the given day-ahead electricity tariff. Moreover, we used flexible charging rates for EVs in this paper given that the day-ahead pricing tariff is also variable. This mode of flexible charging is known as C-F mode [35].

### 6.3. Simulation Results of Proposed Charging Scheme under Demand Response Events

**Figure 6:**
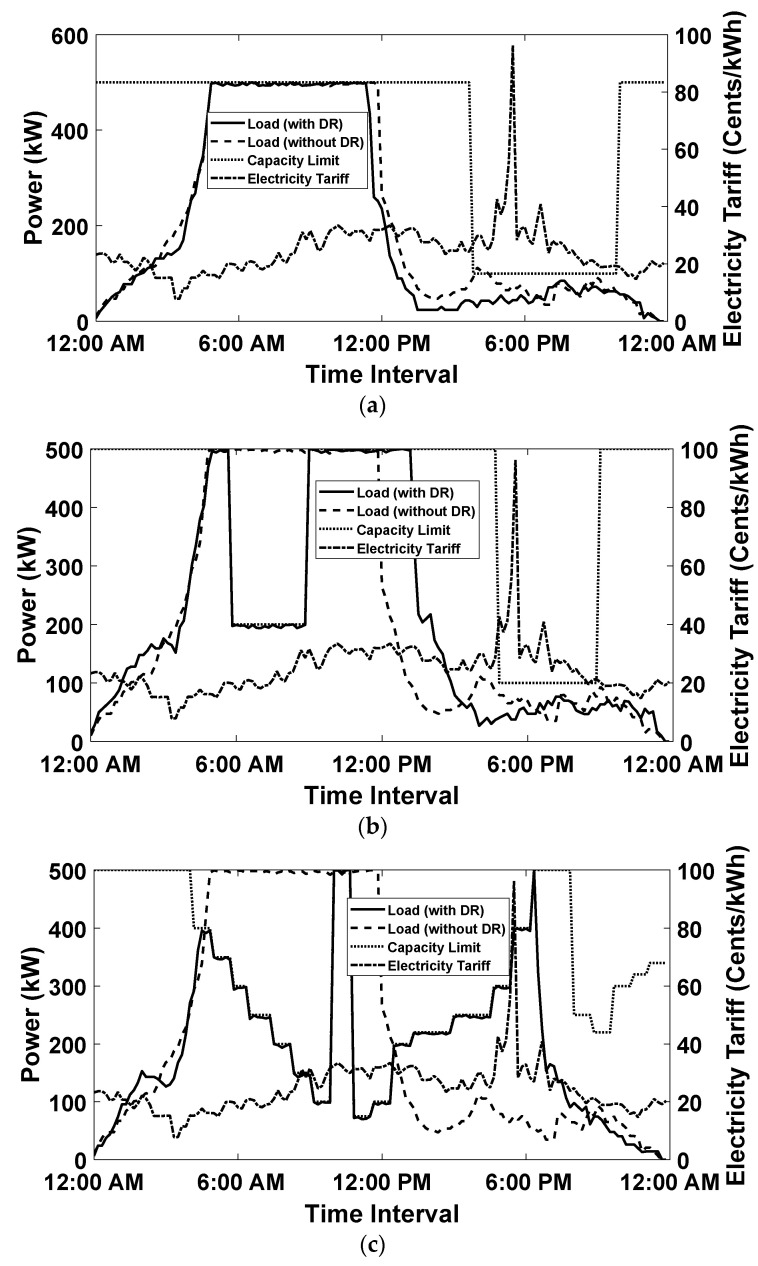
Electric Load Profiles of Smart parking lot with and out DR events. (**a**) Single Constant Load Shedding DR Event (DR1), (**b**) Two Constant Load Shedding DR Event (DR2), (**c**) Variable Load Shedding DR Event (DR3).

**Figure 7:**
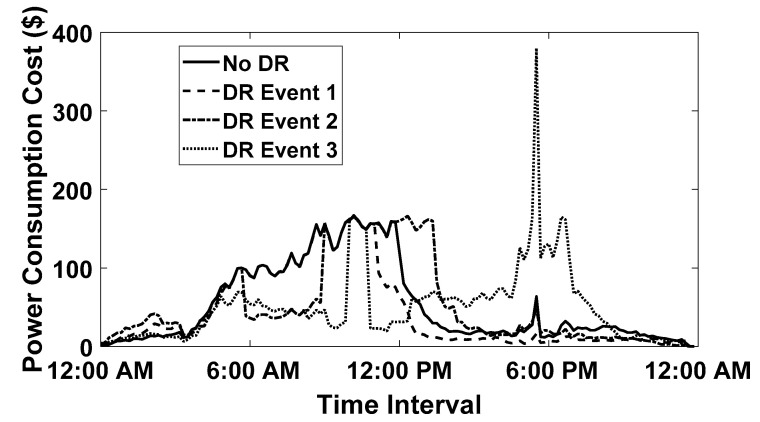
Power Consumption Cost comparison between DR Events.

In order to validate the performance of the proposed On-Off EV charge scheduling technique, three DR events are generated and simulated with the same variable day-ahead electricity tariff illustrated in Figure 5 [21,22,23]. The first DR event (DR1) is a long load shedding DR event generated from 4:00 pm to 10:00 pm, the simulation results of which are presented in Figure 6a. The second DR event (DR2) comprises of short load shedding events from 6:00 am to 9:00 am and from 5:00 pm to 9:00 pm. The simulation results of the second DR event are illustrated in Figure 6b. Meanwhile, the third DR event (DR3) contains two load shedding events that are variable in time, the simulation results of which are depicted in Figure 6c. The results illustrated in Figure 6a–c shows that for all three DR events, the EV charge scheduling arrangement does not exceed the maximum power capacity threshold limit for the total number of EVs (E) equals 500. Moreover, the inclusion of a parameter $\alpha_{p}^{x}$ presented in Equation (3) curtails the optimization process from scheduling the charging of EVs in the high rate tariff intervals to minimize the overall charging cost. Therefore, the charging cost of all three DR events was compared with no DR event and depicted in Figure 7, which shows that under the influence of load shedding events, the overall charging cost for the smart parking lot has not increased in spikes. 

The proposed charging mechanism is compared against the real-valued variable charging power approach in order to verify the capability and effectiveness of the proposed technique. In variable charging rate technique, the objective is to vary the charge rate of an EV attached to the charging pole instead of charging the EV on fixed charging power, so that the charging efficiency of the EV battery must not be affected in a given time interval and the maximum number of arrived EV can be accommodated in the smart parking lot. This condition would be helpful if an empty smart parking pole was available, but we were waiting for an EV to be moved out due to the maximum capacity limit. Moreover, we drop the C-rate of the EV battery from 1 C to 0.5 C in a variable charging rate scheme. For this reason, the EV will take a longer time to charge and stay in the smart parking lot for longer time intervals. However, our proposed LP-based simplified convex relaxation approach is found to outperforms the variable charging rate technique. The comparison is established based on the percentage of electricity cost-saving, the number of fully charged EV over the entire day, and average computational time. The aforementioned comparisons are tabulated for all DR events. 

### 6.4. Comparative Analysis of Proposed IntelligentCharging Scheme with Variable Charging Rate Scheme

In Table 3, the power consumption cost comparison of the proposed algorithm is conducted with the variable charging rate scheme for different numbers of EVs and all DR programs. 

The results make it evident that the proposed scheme is more cost-effective than other techniques. Moreover, In Table 4, we compared the number of EVs fully charged on a given day with a fixed number of charging pole (X=200). Table 4 depicts the improved performance of the proposed algorithm compared to the variable charging rate scheme. 

The performance of the proposed algorithm is also tested against the average SoC of the EVs at the departure time and tabulated in Table 5. The comparison is conducted for different numbers of vehicles E = 100 to 500. It is observed that the proposed technique performs better as the average SoC of an EV leaving the smart parking lot is 97% or above in five different vehicle counts. Moreover, the average time intervals required to achieve final SoC are also computed and compared in Table 6. Again, the proposed technique is clearing, which takes fewer time intervals to charge an EV on average. Therefore, the smart parking lot operator saves time to incorporate a greater number of EVs illustrated in Table 4, where a greater number of EV are charged during course of the entire day. 

The computation time is also deduced for both algorithms on the same machine and listed in Table 7. It is found that the proposed algorithm marginally takes more computational time; however, the time difference is not increased to an alarming situation. 

## 7. Conclusions and Future Work

A real-time and robust EV charging scheme is proposed for EV smart parking lots working under different DR programs. The objective of this proposed scheme is to charge the maximum number of EVs in a day at the minimum possible cost by taking into consideration DR events. The proposed LP and simplified convex relaxation-based on-off EV charging technique is also computationally viable for implementation in real-time scenarios. Moreover, the smart parking lot owner can participate in DR events and be recompensed by the utility after ensuring the variable or constant demand reduction. As soon as the demand limitation is committed for DR, the anticipated optimal EV charging scheduling strategy can start scheduling EVs for intelligent charging such that the overall demand for the load will remain within the constrained of prearranged demand limit. The benefits of the proposed charging scheme are two-fold: (1) the power consumption cost of the smart parking lot is minimized; and (2) the proposed EV intelligent charging technique has a minimal impact on the life cycle of the EV battery. The battery of the EV will always be charged at the maximum and fixed charging rate of its level-II charge standard and on C-rate equals to 1 C. The proposed intelligent charging technique will be tested in future under the presence of on-site renewable power generation sources. The variability of the available renewable power will be an interesting variable to deal in real-time. Finally, state-of-the-art machine learning techniques [36] are increasingly being deployed across a wide range of real-world optimization problems [37]. An interesting direction of future work could be to explore the incorporation of prediction models [38] to further enhance the capabilities of our proposed approach. 

## Figures and Tables

**Table 1:** Notations used in the System Model.

Notation	Definition
Indices	
p	Index for charging pole changing from 1,…,P
x	Index for the number of time interval changing from 1,…,X
I	Index for all charging poles connected with EV at time interval x
$\Pi^{x}$	charging poles indices with respect to charging priority in descending order
Constants	
P	Total number of charging poles
$S_{t}$	Sampling Interval
X	Total Number of Time Intervals
$t_{p}^{arr}$	Arrival time of an EV attached to pole p
$n_{p}^{arr}$	Real-valued arrival time interval of the EV attached to pole p
$t_{p}^{dep}$	Departure time of an EV attached to pole p
$n_{p}^{dep}$	Real-valued departure time interval of the EV attached to pole p
$\gamma_{p}^{x}$	Current State-of-Charge of the EV attached to the pole p
$\gamma^{min},\;\gamma^{max}$	Minimum and maximum boundary limit for State-of-Charge
$C_{p}^{cap}$	Maximum charging capacity of the EV’s battery
$U_{p}^{x}$	Total time intervals required for the charging of EV attached to pole p
$\omega_{p}$	Rank function value of every EV attached to the pole p
$Z_{p}^{max}$	Maximum charging rate of an EV attached to the pole p
$\beta_{min},\;\beta_{max}$	Lowest and highest electricity rates
$Z_{total}$	Total power capacity bound on EV parking lot
$Z_{p}^{max}$	Maximum charging power drawn by the EV attached to the pole p
$C_{p}^{cap}$	Maximum energy storing capacity of the EV attached to the pole p
η	Charging efficiency of the EV
Binary Variables	
$\delta_{p}^{x}$	1, if an EV is attached to pole p at time interval x and 0 otherwise
$k_{p}^{x}$	1, if an EV is attached to pole p at time interval x is charging and 0 otherwise
Continuous Variables	
$r_{p}^{x}$	Weighted charging priority of EV attached to pole p at time interval x
$\alpha_{p}^{x}$	Preference level of pth charging pole to charge attached EV
$\beta^{x}$	Electricity rate at each time interval x
$Z_{DR}^{x}$	Demand Curbing of demand response event
${\hat{k}}_{p}^{y}$	Real-valued charging decision variable

**Table 2:** Battery Capacity, Charge Rate and Division of Electric Vehicles.

EV Types	Battery Capacity (kWh)	Max. Charging Rate at Level II (kW)	Total Percentage of Cars (%)
Mitsubishi i-MiEV	16	3.6	10
Chevy Volt	18	3.6	10
Ford Focus Electric	23	6.6	15
Fiat 500E	24	6.6	15
Kia Soul EV	27	6.6	15
Mercedes B-Class	28	10	5
BMW i-3	33	7.7	5
Volkswagen E-Golf	36	7.2	10
Nissan LEAF	40	6.6	10
Tesla Model-S	100	10	5

**Table 3:** Comparison of Power Consumption Cost ($/day) and saving (in percentage).

	*DR Event*	Number of EV Arrived in a Day (E)				
		100	200	300	400	500
**Variable Charging Rate**	***No DR***	2694	5553	7079	7000	7245
	***DR1***	2597	5776	7081	7343	7385
	***DR2***	2661	5575	7495	7016	7153
	***DR3***	2687	6068	6888	6926	6960
**Proposed**	***No DR***	2600 (↓3.6%)	5457 (↑1.7%)	6655 (↓5.9%)	6668 (↓4.7%)	7028 (↓2.9%)
	***DR1***	2558 (↓1.5%)	5481 (↓5.1%)	6855 (↓3.1%)	7077 (↓3.6%)	7136 (↓3.3%)
	***DR2***	2564 (↓3.6%)	5476 (↓1.7%)	6903 (↓7.8%)	6928 (↓1.2%)	6800 (↓5.0%)
	***DR3***	2637 (↓1.8%)	5872 (↓3.2%)	6933 (↑0.6%)	6988 (↑0.8%)	6873 (↓1.2%)

**Table 4:** Comparison of Number of Fully Charged EVs.

	*DR Events*	Number of EV Arrived in a Day (E)				
		100	200	300	400	500
**Variable Charging Rate**	***No DR***	100	200	203	203	210
	***DR1***	100	200	206	202	214
	***DR2***	100	200	201	207	205
	***DR3***	100	200	200	203	204
**Proposed**	***No DR***	100	200	203	215	216
	***DR1***	100	200	201	214	218
	***DR2***	100	200	201	213	219
	***DR3***	100	200	202	210	217

**Table 5:** Average State-of-Charge (SoC) of the EV’s Battery at departure time.

E	100	200	300	400	500
**Variable Charging Rate**	0.99	0.99	0.94	0.93	0.93
**Proposed**	0.99	0.99	0.98	0.96	0.96

**Table 6:** Comparison for Average Time Intervals required to Achieve Final SoC.

E	100	200	300	400	500
**Variable Charging Rate**	23.02	23.10	20.19	21.12	20.56
**Proposed**	16.20	17.98	17.06	18.56	18.01

**Table 7:** Comparison of Average Computation Time (Seconds) for Algorithms under No DR Event.

E	100	200	300	400	500
**Variable Charging Rate**	5.18	5.21	5.37	5.37	5.40
**Proposed**	5.63	5.73	5.83	5.86	5.77

## References

[1] Egbue O., Long S. (2012). Barriers to widespread adoption of electric vehicles: An analysis of consumer attitudes and perceptions. Energy Policy.

[2] Qian K., Zhou C., Allan M., Yuan Y. (2011). Modeling of load demand due to EV battery charging in distribution systems. IEEE Trans. Power Syst..

[3] Qadir H., Khalid O., Khan M.U., Khan A.U.R., Nawaz R. (2018). An optimal ride sharing recommendation framework for carpooling services. IEEE Access.

[4] Shao S., Pipattanasomporn M., Rahman S. (2012). Grid integration of electric vehicles and demand response with customer choice. IEEE Trans. Smart Grid.

[5] Pipattanasomporn M., Kuzlu M., Rahman S. (2012). An algorithm for intelligent home energy management and demand response analysis. IEEE Trans. Smart Grid.

[6] Zhou S., Wu Z., Li J., Zhang X.-P. (2014). Real-time energy control approach for smart home energy management system. Elect. Power Compon. Syst..

[7] Sajid S., Jawad M., Qureshi M.B., Khan M.U.S., Ali S.M., Khan S.U. A Conditional-Constraint Optimization for Joint Energy Management of Data Center and Electric Vehicle Smart parking lot. Proceedings of the Tenth International Green and Sustainable Computing Conference (IGSC).

[8] Mehmood F., Khan B., Ali S.M., Qureshi M.B., Diver C., Nawaz R. (2020). Multi-Renewable Energy Agent Based Control for Economic Dispatch and Frequency Regulation of Autonomous Renewable Grid. IEEE Access.

[9] Su W., Chow M.-Y. (2012). Computational intelligence-based energy management for a large-scale PHEV/PEV enabled municipal smart parking deck. Appl. Energy.

[10] Rustam M.A., Khan B., Ali S.M., Qureshi M.B., Mehmood C.A., Khan M.U.S., Nawaz R. (2019). An Adaptive Distributed Averaging Integral Control Scheme for Micro-Grids with Renewable Intermittency and Varying Operating Cost. IEEE Access.

[11] Xu Z., Hu Z., Song Y., Zhao W., Zhang Y. (2014). Coordination of PEV charging across multiple aggregators. Appl. Energy.

[12] Huang Q., Jia Q.S., Qiu Z., Guan X., Deconinck G. (2015). Matching EV charging load with uncertain wind: A simulation-based policy improvement approach. IEEE Trans. Smart Grid.

[13] Kuran M.S., Viana A.C., Iannone L., Kofman D., Mermoud G., Vasseur J.P. (2015). A smart parking lot management system for scheduling the recharging of electric vehicles. IEEE Trans. Smart Grid.

[14] Shafie-khah M., Heydarian-Forushani E., Osório G.J., Gil F.A., Aghaei J., Barani M., Catalão J.P. (2016). Optimal Behavior of Electric Vehicle Smart Parking Lots as Demand Response Aggregation Agents. IEEE Trans. Smart Grid.

[15] He Y., Venkatesh B., Guan L. (2012). Optimal scheduling for charging and discharging of electric vehicles. IEEE Trans. Smart Grid.

[16] Jian L., Zhu X., Shao Z., Niu S., Chan C.C. (2014). A scenario of vehicle-to-grid implementation and its double-layer optimal charging strategy for minimizing load variance within regional smart grids. Energy Convers. Manag..

[17] Zhang L., Li Y. (2016). A game-theoretic approach to optimal scheduling of smart parking lot electric vehicle charging. IEEE Trans. Veh. Technol..

[18] Ansari M., Al-Awami A.T., Sortomme E., Abido M.A. (2015). Coordinated bidding of ancillary services for vehicle-to-grid using fuzzy optimization. IEEE Trans. Smart Grid.

[19] Jin C., Tang J., Ghosh P. (2013). Optimizing electric vehicle charging with energy storage in the electricity market. IEEE Trans. Smart Grid.

[20] Han S., Han S., Sezaki K. (2010). Development of an optimal vehicle-to-grid aggregator for frequency regulation. IEEE Trans. Smart Grid.

[21] Shafie-khah M., Heydarian-Forushani E., Golshan M.E., Siano P., Moghaddam M.P., El-Eslami M.K., Catalão J.P. (2016). Optimal trading of plug-in electric vehicle aggregation agents in a market environment for sustainability. Appl. Energy.

[22] Yazdani-Damavandi M., Moghaddam M.P., Haghifam M., Shafie-khah M., Catalão J.P. (2016). Modeling Operational Behavior of Plug-in Electric Vehicles’ Smart parking Lot in Multienergy Systems. IEEE Trans. Smart Grid.

[23] Škugor B., Deur J. (2015). Dynamic programming-based optimisation of charging an electric vehicle fleet system represented by an aggregate battery model. Energy.

[24] Lausenhammer W., Engel D., Green R. (2016). Utilizing capabilities of plug in electric vehicles with a new demand response optimization software framework: Okeanos. Int. J. Electr. Power Energy Syst..

[25] Tan Z., Yang P., Nehorai A. (2014). An optimal and distributed demand response strategy with electric vehicles in the smart grid. IEEE Trans. Smart Grid.

[26] Rassaei F., Soh W.-S., Chua K.-C. (2015). Demand response for residential electric vehicles with random usage patterns in smart grids. IEEE Trans. Sustain. Energy.

[27] Yao L., Damiran Z., Lim W.H. (2017). Optimal Charging and Discharging Scheduling for Electric Vehicles in a Smart parking Station with Photovoltaic System and Energy Storage System. Energies.

[28] Cao Y., Kaiwartya O., Wang R., Jiang T., Cao Y., Aslam N., Sexton G. (2017). Toward Efficient, Scalable, and Coordinated On-the-Move EV Charging Management. IEEE Wirel. Commun..

[29] Cao Y., Jiang T., Kaiwartya O., Sun H., Zhou H., Wang R. (2019). Toward Pre-Empted EV Charging Recommendation Through V2V-Based Reservation System. IEEE Trans. Syst. Man. Cybern. Syst..

[30] ERCOT Meteorological Data of Texas from National Estuarine Research Reserve System [Online]. http://cdmo.baruch.sc.edu/get/export.cfm/.

[31] Jawad M., Qureshi M.B., Khan U., Ali S.M., Mehmood A., Khan B., Khan S.U. (2018). A robust Optimization Technique for Energy Cost Minimization of Cloud Data Centers. IEEE Trans. Cloud Comput..

[32] Şengör İ., Erdinç O., Yener B., Taşcıkaraoğlu A., Catalão J.P. (2019). Optimal energy management of EV smart parking lots under peak load reduction-based DR programs considering uncertainty. IEEE Trans. Sustain. Energy.

[33] Jawad M., Qureshi M.B., Nadeem A., Ali S.M., Shabbir N., Rafiq M.N. Bi-Directional Nano Grid Design for Organizations with Plug-In Electric Vehicle Charging at Workplace. Proceedings of the 2018 IEEE International Conference on Electro./Information Technology (EIT).

[34] Nadeem A., Rafiq M.N., Qureshi M.B., Jawad M. Joint Power Management of Telecom Exchanges and Electric Vehicles Using Hybrid AC-DC Microgrid. Proceedings of the International Conference on Frontiers of Information Technology (FIT).

[35] Mao T., Zhang X., Zhou B. (2019). Intelligent Energy Management Algorithms for EV-charging Scheduling with Consideration of Multiple EV Charging Modes. Energies.

[36] Yunus R., Arif O., Afzal H., Amjad M.F., Abbas H., Bokhari H.N., Haider S.T., Zafar N., Nawaz R. (2019). A Framework to Estimate the Nutritional Value of Food in Real Time Using Deep Learning Techniques. IEEE Access.

[37] Anwaar F., Iltaf N., Afzal H., Nawaz R. (2018). HRS-CE: A hybrid framework to integrate content embeddings in recommender systems for cold start items. J. Comput. Sci..

[38] Ayyaz S., Qamar U., Nawaz R. (2018). HCF-CRS: A Hybrid content based fuzzy conformal recommender system for providing recommendations with confidence. PLoS ONE.

